# 

**Published:** 1866-03

**Authors:** 


CONTENTS FOR MARCH.

ORIGINAL CONTRIBUTIONS.

On the Cause of Intermittent Fever. By Henry M. Lyman, M. D......p. 97

Clinical Lectures on Diseases of the Eye. By E. L. Holmes, M. D.......104

On the Respiratory Function in Pneumonia. Report of Cases. By Dr. J. P. Ross.........................................................109

Cases Reported to the Chicago Medical Society. By Dr. Charles G. Smith.........................................................114

PROCEEDINGS OF MEDICAL SOCIETIES.

The Chicago Medical Society.........................................................117

Rock River Union Medical Society.........................................................120

SELECTED ARTICLES.

Last Illness of Valentine Mott, M. D.........................................................122

*Surgery*—Endoscopy.........................................................126

EDITORIAL CORRESPONDENCE.........................................................130

EDITORIAL.........................................................133

*Book Notices*—The Physiology of Man: Designed to represent the existing state of Physiological Science as applied to the Functions of the Human Body—Chloroform and its Administration—The Principles and Practice of Medicine.

Chicago Medical College—Death of Dr. I. P. Lynn, etc.

---

THE CHICAGO

MEDICAL JOURNAL.

